# Saudi Arabian road accident mortality and traffic safety interventions dataset (2010–2020)

**DOI:** 10.1016/j.dib.2022.108502

**Published:** 2022-07-30

**Authors:** Syed Arshad Raza, Atiq W. Siddiqui, Farhan Muhammad Butt, Muhammad Ather Elahi, Khurram Shahid Minhas

**Affiliations:** a College of Business Administration, Imam Abdulrahman Bin Faisal University, Saudi Arabia; b Department of Community Development, Lee County, Florida, USA; c Department of Transportation and Traffic Engineering, College of Engineering, Imam Abdulrahman Bin Faisal University, Saudi Arabia

**Keywords:** Traffic safety, road safety, road accident mortalities, safety interventions, global road safety plan

## Abstract

Increased traffic volumes worldwide have resulted in an increased number of road accident injuries and mortalities. This global phenomenon motivated the United Nations (UN) to initiate a decade-long global road safety plan in 2010. In response, Saudi Arabia concurrently initiated a comprehensive road safety program, supported by detailed and comprehensive road safety data for the Eastern Province (EP) of Saudi Arabia. The contributed **EP-Traffic-Mortality-and-Policy-Interventions Dataset** provides multidimensional road safety data for 2010–2020 via two primary and five secondary data subsets. The first primary subset provides road accident mortality data. The five secondary data subsets reflect road accident mortalities at different time scales and administrative (provincial or governorate) levels. The second primary subset provides details of traffic safety policy interventions implemented during the same period. Researchers and policymakers can use this comprehensive dataset to study accident mortality patterns across various geospatial and time scales and analyze the effectiveness of policies intended to mitigate road accident mortalities.

## Specifications Table

**Table:** 

Subject	Transportation Management, Safety Research, Planning and Development
Specific subject area	Road Safety, Policy Analysis, Transportation Planning
Type of data	Tabular Data Figure Map Textual Descriptions
How the data were acquired	The datasets were obtained from 1) the Ministry of Health data warehouse, 2) the Ministry of Interior's Traffic and Security Police Department's database 3) Saudi Red Crescent Authority's database, 4) major hospitals, trauma centers, and Morgues databases; and 5) the documentation of the Eastern Province Traffic Safety Council.
Data format	Two primary datasets are raw Five secondary datasets (derived from the above two primary datasets) are filtered and processed
Description of data collection	The first primary dataset on road accident mortalities was collected from sources 1-4. This data was checked for data completeness and accuracy. The second primary dataset was derived from source 5.
Data source location	Sources 1-5 identified above, located in the Eastern Province of Saudi Arabia
Data accessibility	Repository name: Mendeley.com Data identification number: doi:10.17632/f5t4kvmn8g.2 Direct URL to data: https://data.mendeley.com/datasets/f5t4kvmn8g/2 Instructions for accessing these data: The data is stored in EP-traffic-mortality-and-policy-interventions-dataset.xlsx file
Related research article	This article is submitted with: A.W. Siddiqui, S.A. Raza, M.A. Elahi, K.S. Minhas, & F.M. Butt. Temporal impacts of road safety interventions: A structural-shifts-based method for road accident mortality analysis, Accident Analysis & Prevention 174 (2022) 106767.

## Value of the Data

• Descriptive analytics can be employed to study accident mortality patterns.
• The datasets on accident mortalities and safety interventions can jointly be used to analyze and improve road safety awareness programs and policy effectiveness.
• The secondary datasets on accident mortalities are useful for analyzing geospatial and temporal variations.
• The secondary datasets on accident mortalities can be employed to compare road safety policy performance in other locations of comparable geospatial characteristics.
• Machine learning and forecasting researchers can use the datasets for accident mortality prediction.

## 1 Data Description

The EP-Traffic-Mortality-and-Policy-Interventions Dataset is publicly available as a single Excel file (**EP-traffic-mortality-and-policy-interventions-dataset.xlsx**) at Mendeley website (Link: https://data.mendeley.com/datasets/f5t4kvmn8g/2). The file includes several individual Excel sheets that contain: (1) the primary data subsets for road accident mortality and traffic safety policy interventions data, (2) the secondary data subsets that reflect road accident mortalities across different time scales and administrative (provincial or governorate) levels, (3) related supplementary information obtained from external sources, and (4) an initial description of the dataset organization and closing acknowledgments regarding dataset development. Table 1 summarizes the file contents for the EP-Traffic-Mortality-and-Policy-Interventions Dataset.

**Table 1:** File contents of the EP-Traffic-Mortality-and-Policy-Interventions Dataset.

EXCEL SHEET	DATASET	TABLE & TABLE FIELDS | FIGURES
DATA DESCRIPTION	-	**Table 1.** Dataset Description **Table 2.** File Organization 1. Data Sheet (with links) 2. Short Description

RAW ACCIDENT MORTALITY DATA	Traffic Accident Mortality Primary dataset	**Table 1.** Traffic Accident Mortality Primary dataset 1. death date (Gregorian) 2. death date (Hijri) 3. Place of Death 4. Age (Day/Month/Year) 5. Gender 6. Nationality 7. Hospital Code 8. City 9. Governorate 10. Population **Table 2.** Data Source

PERIODIC ACCIDENT MORTALITY DATA (PROVINCE-LEVEL)	Periodic Traffic Accident Mortality Secondary datasets (Province-Level)	**Table 1.** Yearly Mortality Time Series **Table 2.** Monthly Mortality Time Series **Table 3**. Weekly Mortality Time Series 1. Year | Month-Year | Week-Year (Tables 1-3) 2. Deaths **Table 4.** Data Source

PERIODIC ACCIDENT MORTALITY DATA (GOVERNORATE-LEVEL)	Periodic Traffic Accident Mortality Secondary datasets (Governorate-Level)	**Table 1.** Yearly Mortality Time Series (Governorate-Level) **Table 2.** Monthly Mortality Time Series (Governorate-Level) 1. Year | Month (Tables 1-2 respectively) 2. 12 columns reporting periodic deaths for 11 Governorates. Last column is for unreported locations **Table 3.** Data Source

POLICY INTERVENTIONS TO PREVENT ROAD ACCIDENTS	Policy Interventions: 2010-2020	**Table 1.** Policy Interventions 1. Date 2. Intervention 3. Description 4. Policy Recommendation/Implementation 5. Region Applied 6. Source

EP MAP	Eastern Province Demographics	**Figure.** Saudi Arabia map with Eastern Province Highlighted **Table 1.** Eastern Province: KSA **Table 2.** Data Source

GOVERNORATE & CITIES	Eastern Province governorates Demographics	**Figure.** Saudi Arabia map with Eastern Province Highlighted **Table 1.** Governorates Location & Population **Table 2.** Cities Location & Population **Table 3.** Data Source **Figure.** Saudi Arabia map with Eastern Province Governorates Highlighted

CREDITS	Authors Details	**Table 1.** Author Details 1. Researcher 2. Affiliation 3. Email

The first sheet in the Excel file, **DATA DESCRIPTION**, contains two tables. In these tables, Table 1 provides a general description of the consolidated dataset, while Table 2 provides a more detailed description of file organization, including sheet names (with hyperlinks) and available dataset(s). Data sources are also reported on each of the respective sheets.

The second sheet in the Excel file, **RAW ACCIDENT MORTALITY DATA,** contains two tables. Here, Table 1 reports primary data on accident mortality in the Eastern Province of Saudi Arabia from September 7, 2009, to January 1, 2010. The total number of reported road accident mortality cases in this dataset is 7,351. Ten attributes were recorded for each case, including the victim's age, gender, nationality, date of death (two calendar systems), location of death (city/governorate), place of death (before reaching hospital/in-hospital) and the related hospital code. Table 2 identifies the sources of this data.

**Fig. 1:**
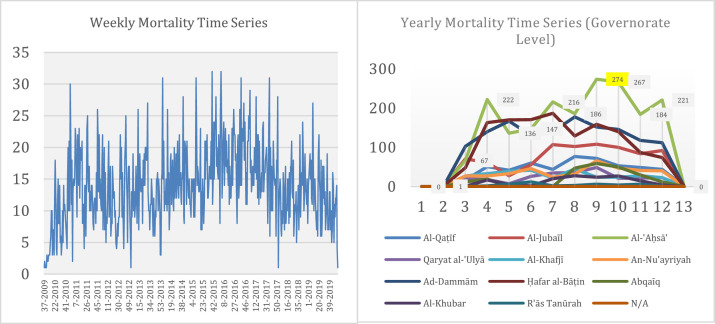
Road accident mortality time-series data visualization (Sep 2009–Jan 2020): Weekly mortalities at the provincial level (left) and yearly mortalities at the governorate level (right).

The subsequent two sheets in the Excel file, **PERIODIC ACCIDENT MORTALITY DATA (PROVINCE-LEVEL)** and **PERIODIC ACCIDENT MORTALITY DATA (GOVERNORATE-LEVEL)**, reflect derived datasets obtained from the **RAW ACCIDENT MORTALITY DATA** dataset discussed above. The first sheet provides the yearly, monthly, and weekly mortalities at the provincial level as a time series to support visualization. The second sheet provides yearly and monthly mortalities at the governorate level with geospatial separation as a time series. Note that the Eastern Province of Saudi Arabia has 11 governorates, each overseeing multiple cities. Weekly data was not provided at the governorate level because of the high data sparsity at that temporal resolution. To provide a general understanding of the potential temporal and geospatial scenarios supported by this dataset, Fig. 1 depicts the weekly and yearly mortalities at the provincial and governorate levels, respectively, as time series visualizations. Varying mortality levels are visible at provincial and governorates levels and among governorates. Note that the 31 records (0.42% of 7,351 records) with missing locational information (e.g., city/place of death) were separately identified in the governorate-level visualization as N/A.

To support the accurate interpretation of the road accident mortality data, the Excel file also includes supplemental information in two separate sheets regarding geographical and demographical details of Saudi Arabia's Eastern Province and its governorates (**EP MAP** & **GOVERNORATE & CITIES)**. Two separate maps of the EP are provided: an overview map in the first sheet that highlights the size of the EP (covering 710,000 km²) and a more detailed map in the second sheet that depicts the locations of all governorates within the EP. The tabular data contained in these sheets detail the total population of the EP (8,260,066 in the 2010 census), the latitude and longitude values of various geographic locations and the populations of the governorates and their major cities. The relevant government data sources are also reported on both sheets.

The remaining substantive content in the Excel file is the **POLICY INTERVENTIONS TO PREVENT ROAD ACCIDENTS** sheet. This data subset reports policy interventions enacted during the same decade-long analysis period (2010–2020) and includes intervention enactment dates, features, development details (where available), policy recommendation(s), implementation details (including the scope or region of implementation), and related validation studies. This sheet also identifies the source government agency that provided the policy/intervention details. These details are also summarized tabularly in the Excel file.

## 2 Experimental Design, Materials and Methods

For complex data collection processes with various overlapping data sources and different data recording methods, data correctness, completeness, and consistency are of utmost importance for the validity of the dataset. With these three quality-metrics in mind, we developed a systematic approach for data collection when creating the EP-Traffic-Mortality-and-Policy-Interventions Dataset. To ensure data completeness, we initially identified all relevant stakeholders and their respective program roles, data sources, and data sharing mechanisms. Target data was then collected from all relevant sources and processed to eliminate any incompleteness or inconsistencies. These steps are detailed below.

**Fig. 2:**
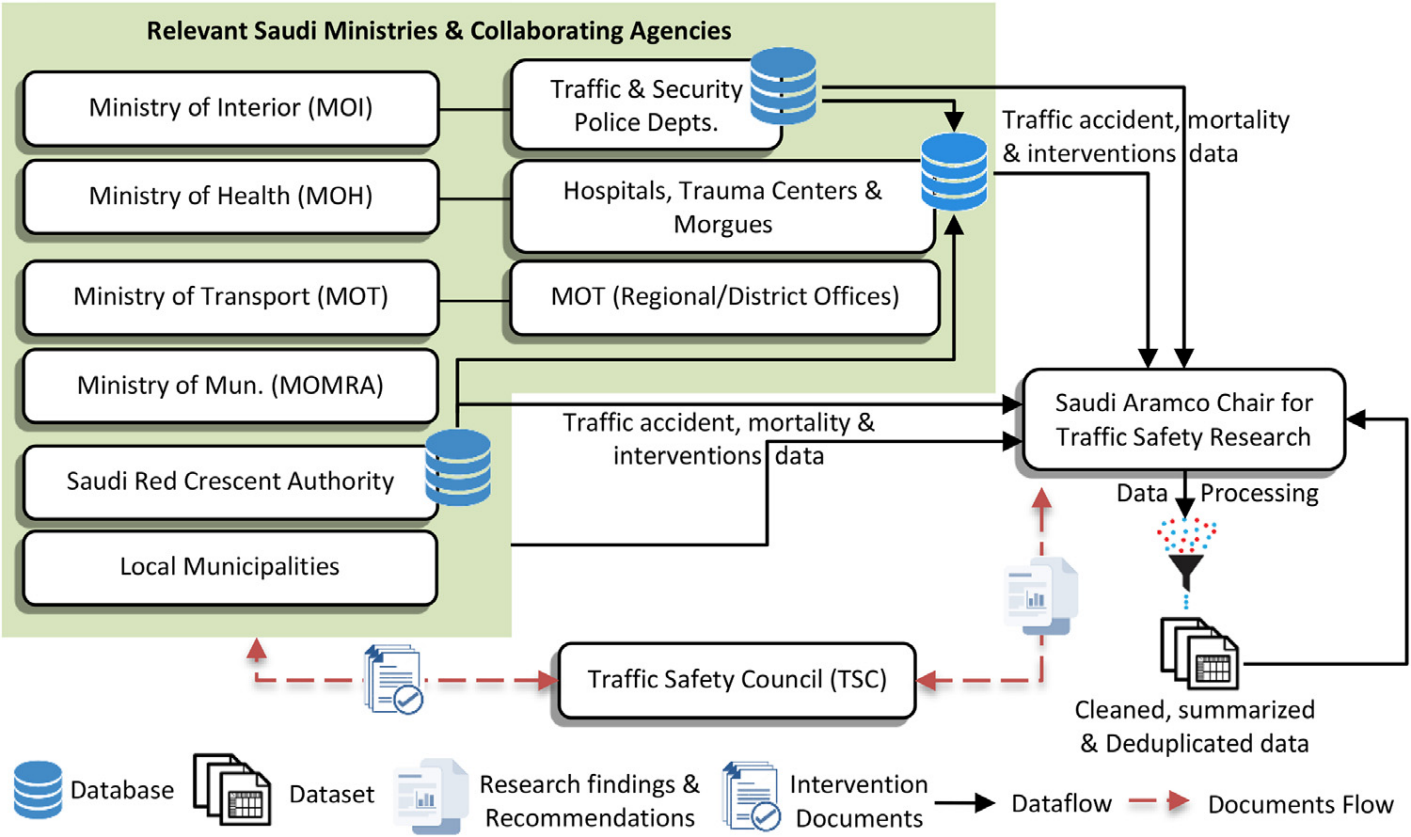
Relevant road safety stakeholders and their data sources and sharing mechanisms.

**Stakeholder identification.** The first step in developing the EP-Traffic-Mortality-and-Policy-Interventions Dataset was to identify all relevant road safety stakeholders. The primary stakeholder identified during this task was the Eastern Province Traffic Safety Council (TSC), which is a centralized program coordination body led by the provincial governor. Its mission is to formulate traffic safety policies and coordinate and monitor participating stakeholders. The TSC is also mandated to contact any other related agency or entity within the Eastern Province for specific traffic safety related tasks or projects. Other key governmental stakeholders involved in road safety include the Ministry of Transport (MOT), Ministry of Interior (MOI), Ministry of Health (MOH), Ministry of Municipalities and Rural Authorities (MOMRA), Saudi Red Crescent Authority (SRC), emergency and trauma centers and morgues, public transportation agencies, and local municipalities. These collective stakeholders were identified because of their direct role in managing and maintaining the road infrastructure, controlling road traffic, enforcing safety rules, and managing emergency response services. The Saudi Traffic Safety Association (SALAMAH) was also identified as an important stakeholder. Serving in a public awareness role for the TSC, SALAMAH promotes traffic safety culture via awareness campaigns in coordination with the above stakeholders, traffic safety professionals, and interested community members. Finally, the Saudi Aramco Chair for Traffic Safety Research was identified as a key stakeholder because of its role in studying and validating the effectiveness of safety interventions, proposing improvements, and supporting implementations through consultation. Fig. 2 depicts the stakeholder interactions, as well as their respective data sources and data sharing mechanisms (detailed below).

**Data sources and data sharing mechanisms.** After identifying all relevant road safety stakeholders, the next step in developing the EP-Traffic-Mortality-and-Policy-Interventions Dataset was to identify all corresponding data sources and data sharing mechanisms. For accident mortality data, the Ministry of Interior's Traffic and Security Police Department's database was identified as a key data source. Local Traffic and Security Police Department personnel are typically the first responders to a road accident and are responsible for reporting any injuries and on-scene deaths. A second key data source for accident mortality data included the Saudi Red Crescent Authority (SRC) database, which includes records of accident cases it handles. In coordination with the local police agency, the SRC is responsible for transporting persons involved in a road accident to government-designated trauma centers, hospitals, or the morgue if an on-scene or en route death occurs. Finally, hospitals maintain their own local databases that contain details of road accident victims. These three databases were aggregated under the Ministry of Health (MOH) to form the Province-wide central data warehouse—the EP-Traffic-Mortality-and-Policy-Interventions Dataset. The Eastern Province Traffic Safety Council (TSC) coordinates with the authorities responsible for these three databases to facilitate communication and data acquisition for the Saudi Aramco Chair for Traffic Safety Research. While the consolidated data was obtained to validate data completeness and accuracy, the original datasets from the first two original sources were also obtained. Following thorough cleansing and validation of the combined data by the Saudi Aramco Chair for Traffic Safety Research, various analyses are performed, and results are reported to the TSC for further action (see Fig. 2).

For data regarding traffic safety policy interventions, the Eastern Province TSC was identified as a key data source because of its role in developing, coordinating, and implementing traffic safety strategies, policies, interventions, awareness campaigns, and research programs. The TSC, led by the provincial governor, is responsible for investigating all critical traffic safety issues, devising mitigation tasks in coordination with other stakeholders, and subsequently delegating these tasks to support operationalization. While TSC served as the primary source to support the complete development of the EP-Traffic-Mortality-and-Policy-Interventions Dataset, information regarding intervention developments and features, operationalizations and timelines, interlinkages, and validation studies was gathered directly from the respective stakeholders.

**Data cleansing and validation.** Because the data used to develop the EP-Traffic-Mortality-and-Policy-Interventions Dataset was obtained from various sources, the data aggregation process required systematic validation to ensure the data's correctness, completeness, and consistency.

Before being consolidated in the combined dataset, the raw road accident mortality data obtained from the three data sources from September 2009 to January 2020 was checked for any glaring issues, such as missing data, duplication, and inconsistencies. The initial 42 (0.51% of 7,351 records) duplicated records were deduplicated. Several typos in specific fields (e.g., nationality, city names, etc.) were also detected and corrected using Excel's data duplication and conditional formatting features. A total of 31 records (0.42% of 7,351 records) were missing locational information (e.g., city/place of death). Although this missing data was not able to be corrected, the impact on the combined dataset's quality was thought to be minimal because the number of records with missing data was small and the most recent record with missing data was from September 2010. Finally, any necessary field conversions (e.g., date conversions from Hijri to Gregorian) were performed to maintain consistency across the dataset. For the traffic safety policy intervention data, information was validated directly by the respective stakeholders as required.

Following consolidation, the EP-Traffic-Mortality-and-Policy-Interventions Dataset was subsequently used to derive two primary and five secondary subsets of data. The first primary and five secondary data subsets provide road accident mortality data. The five secondary data subsets reflect different time scales and administrative (provincial or governorate) levels. The second primary data subset provides corresponding traffic safety policy interventions data. Siddiqui et al. [1,2] demonstrate the applicability of data in two different research contexts.

## Ethics Statements

The data reported in this paper was obtained directly from the sources mentioned and did not involve any experiments on humans. Moreover, the secured data neither contained any personal information about the deceased nor any information about the hospitals where they were received or kept after the accident. Hence, no ethics approval was needed for this study.

## CRediT authorship contribution statement

**Syed Arshad Raza:** Data curation, Writing – original draft, Visualization. **Atiq W. Siddiqui:** Data curation, Writing – original draft, Visualization. **Farhan Muhammad Butt:** Resources, Writing – review & editing. **Muhammad Ather Elahi:** Validation, Writing – original draft. **Khurram Shahid Minhas:** Resources, Writing – review & editing.

## Declaration of Competing Interest

The authors declare that they have no known competing financial interests or personal relationships that could have appeared to influence the work reported in this paper.

## Data Availability

Saudi Arabian Road Accident Mortality and Traffic Safety Interventions Dataset (2010–2020) (Original data) (Mendeley Data).
